# High Seroprevalence of Antibodies against Spotted Fever and Scrub Typhus Bacteria in Patients with Febrile Illness, Kenya

**DOI:** 10.3201/eid2104.141387

**Published:** 2015-04

**Authors:** Jacqueline W. Thiga, Beth K. Mutai, Wurapa K. Eyako, Zipporah Ng’ang’a, Ju Jiang, Allen L. Richards, John N. Waitumbi

**Affiliations:** Walter Reed Project/Kenya Medical Research Institute, Kisumu, Kenya (J.W. Thiga, B.K. Mutai, W.K. Eyako, J.N. Waitumbi);; Jomo Kenyatta University of Agriculture and Technology, Itromid, Nairobi, Kenya (J.W. Thiga, Z. Ng’ang’a);; Naval Medical Research Center, Silver Spring, Maryland, USA (J. Jiang, A.L. Richards)

**Keywords:** Rickettsia, spotted fever, typhus fever, scrub typhus, seroprevalence, bacteria, rickettsial infections, Kenya

## Abstract

Serum samples from patients in Kenya with febrile illnesses were screened for antibodies against bacteria that cause spotted fever, typhus, and scrub typhus. Seroprevalence was 10% for spotted fever group, <1% for typhus group, and 5% for scrub typhus group. Results should help clinicians expand their list of differential diagnoses for undifferentiated fevers.

Rickettsioses are zoonoses that are increasingly being recognized as noteworthy infectious diseases (*1*). They are caused by bacteria of the genera *Rickettsia* and *Orientia*, which are small, gram-negative, obligate intracellular bacteria that are transmitted to humans through bites of infected arthropod vectors, such as fleas, mites, ticks, and lice. These bacteria are able to invade various host cells, including vascular endothelium, causing characteristic symptoms such as rash and petecchial hemorrhages. The genus *Rickettsia* is divided into 2 main biogroups: spotted fever group (SFG) and typhus group (TG). The scrub typhus group (STG) previously belonged to the genus *Rickettsia* but now belongs to the genus *Orientia* (*2*), which consists of 2 species: *O. tsutsugamushi* and *O. chuto* (*3*).

The past 5 years have seen concerted efforts to understand the etiology of undifferentiated febrile illnesses, a group of diseases that includes rickettsioses. These efforts have confirmed the occurrence of infections with *R. felis*, transmitted mainly by the cat flea (*Ctenocephalides felis felis*), as a common cause of fever in rural areas (*4*–*6*). Studies have also shown the preponderance of *R. africae*, the causative agent of African tick-bite fever, as well as *R. conorii* and *R. aeschlimannii*, in different ecoregions of Kenya (*7*). The study reported here is part of a broader study aimed at identifying pathogens or their surrogates, such as immunoglobulins, in patients with febrile illnesses. It is hoped that these types of reports will help local clinicians expand their list of differential diagnoses for undifferentiated fevers.

## The Study

The study protocol was approved by the Ethical Review Committee of the Kenya Medical Research Institute (SSC #1282) and the Walter Reed Army Research Institute’s Human Subject Protection Board (WRAIR HSPB #1402). All patients provided informed consent.

Serum samples were collected from patients with fever (>38°C) at 8 hospitals in 6 ecoregions of Kenya (Technical Appendix Figure 1). The samples were screened for IgG against whole-cell antigens of *R. conorii* for SFG, *R. typhi* for TG, and Karp and Gilliam strains of *O. tsutsugumishi* for STG as previously described (*8*,*9*). Serum samples that were reactive at 1:100 dilutions were further titrated by using 4-fold dilutions to 1:6,400.

**Figure 1:**
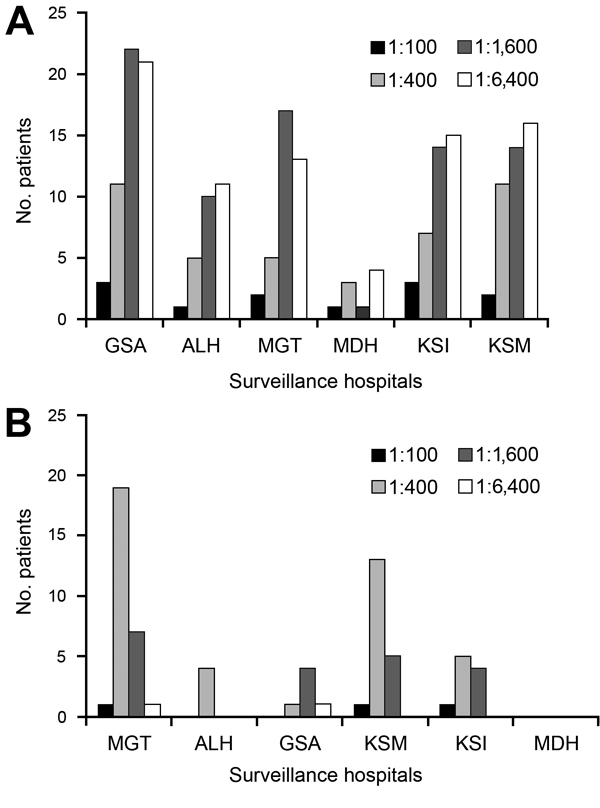
Distribution of titers to spotted fever (SFG) and scrub typhus (STG) groups in patients recruited in various surveillance hospitals. A) For SFG, Garissa District Hospital (GSA), in semiarid northeastern Kenya, had more patients with higher titers compared with Alupe District Hospital (ALH), on the Kenya-Uganda border; Marigat District Hospital (MGT), on the floor of the Rift Valley; Malindi District Hospital (MDH), on the Indian Ocean coast; Kisii District Hospital (KSI), in the Kisii highlands; and Kisumu District Hospital and Obama Children Hospital (KSM), on the Lake Victoria basin. B) For STG, MGT had the most patients with titers of 1:400 and 1:1,600 compared with ALH, MDH, KSI, and KSM.

Because scrub typhus has not been reported in Kenya, Western blot was performed to confirm specificity of the reactive serum samples, essentially as described before (*5*). For Western blot, 0.06 μg/well of the Otr47b antigen was applied to a 10% sodium dodecyl sulfate–polyacrylamide gel and separated by electrophoresis. The proteins were transferred to a nitrocellulose membrane (Invitrogen, Carlsbad, CA, USA). After blocking nonspecific binding at 4°C in 10% skim milk (Difco Becton, Dickinson, Franklin Lakes, NJ, USA), lanes of migrated Otr47b antigens were probed with serum samples that had titers >1:1,600. IgG against Otr47b antigen was detected by a horseradish peroxidase–conjugated anti-human IgG (Kirkegaard & Perry Laboratories, Gaithersburg, MD, USA) at a 1:25,000 dilution, signal visualized by chemiluminescence, and acquired on Kodak X-Ray Film (Carestream Health Inc., Toronto, Ontario, Canada).

A total of 2,225 patients 1–72 years of age (mean age 5 years) were enrolled in the study. There was no difference in the male:female ratio across age groups. Overall, 212 (10%) febrile patients were seropositive for SFG. A substantially higher prevalence rate was seen in Garissa (57/226, 25%) than in Alupe (27/176, 15%), Marigat (37/320, 12%), Malindi (9/102, 9%), Kisii (39/656, 6%), or Kisumu (43/745, 6%) (p<0.05) (Technical Appendix Figure 2).

**Figure 2:**
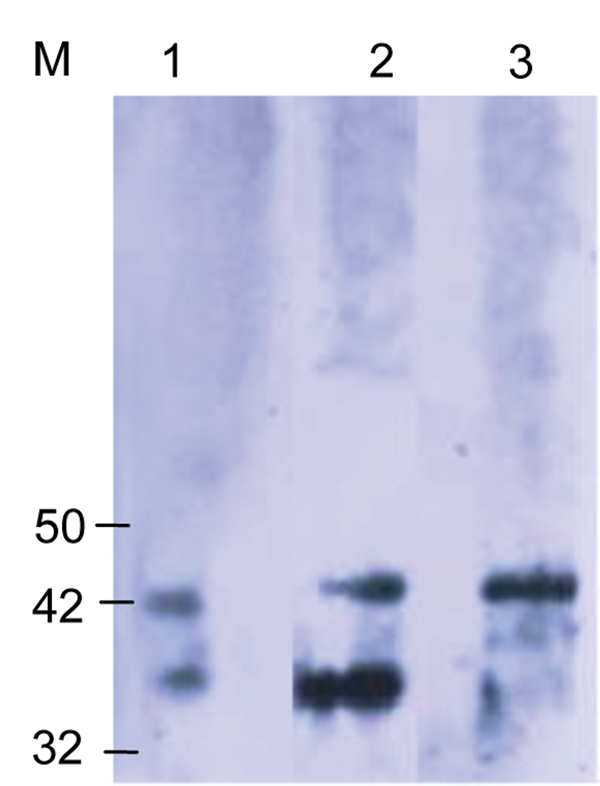
Western blot analysis using the *Orientia* spp.–specific antigen (Otr47b). Twenty scrub typhus reactive serum samples at a titer ≥1:6,000 were used. Negative controls were serum samples that were reactive to spotted fever and typhus group antigens. The scrub typhus reactive serum samples recognized the Otr47b antigen (lanes 2 and 3), but the spotted fever group and typhus group reactive serum samples did not (data not shown). Lane 1 was probed with a positive control serum sample from an earlier scrub typhus outbreak study (*5*). M, molecular mass standard, kDa.

In all regions, most SFG-seropositive patients had titers >1:1,600 (38%), with the highest numbers coming from Garissa (29%, n = 23), followed by Kisumu (18%, n = 16), and Malindi (5%, n = 4) (Figure 1, panel A). Only 4/1,611 (<1%) febrile patients were seropositive for TG: 3 patients in Malindi and 1 in Kisumu. Antibodies against STG rickettsiae were detected in 67/1401 (5%) febrile patients. The highest prevalence was seen in Marigat District Hospital (28/238, 12%), followed by Alupe Sub-District Hospital (4/68, 6%), Garissa (6/134, 5%), Kisumu (19/464, 4%), and Kisii (10/458, 2%) (p<0.05) (Technical Appendix Figure 3). Most STG patients had titers of 1:400 (62%), with the highest coming from Marigat (107/238, 45%) and Kisumu (142/458, 31%) (Figure 1, panel B). Western blot analysis confirmed reactivity of STG serum samples to *O. tsutsugumishi* antigen (Figure 2).

Table shows the prevalence of SFG and STG antibodies by patient age, sex, and animal contact. Female patients were 1.88 times more likely to be exposed to STG than male patients (p = 0.0169), unlike with SFG. Seroprevalence for SFG and STG increased with patients’ age (p<0.05). Having camels and dogs was positively associated with SFG (p<0.05) and having goats with STG (p<0.05).

**Table:** Demographic characteristics of febrile patients tested for seropositivity for SFG and STG rickettsioses, Kenya*

Characteristic	SFG, no. positive/no. tested (%)	OR (95% CI)	STG, no. positive/no. tested (%)	OR (95% CI)
Sex				
F	96/1,094 (9)	1.0	43/694 (6)	1.0
M	116/1,131 (10)	1.2 (0.9–1.6)	24/707 (3)	0.5 (0.3–0.9†*
Age, y				
<5	41/1,107 (4)	1.0	17/687 (3)	1.0
5–12	62/622 (10)	2.9 (1.9–4.4)†	29/423 (6)	2.9 (1.5–5.7)
13–26	63/290 (22)	7.2 (4.7–11.2)†	10/ 166 (6)	2.5 (1.0–6.0)†
>26	46/206 (22)	7.5 (4.6–12.1)†	11/125 (9)	3.8 (1.6–8.8)†
Animal contact				
Goats				
No contact	205/2,188 (9)	1.0	60/1,372 (4)	1.0
Contact	7/37 (19)	2.3 (0.8– 5.3)	7/29 (24)	7.0 (2.4–17.7)†
Cows				
No contact	207/2,187 (10)	1.0	65/1,377 (5)	1.0
Contact	5/38 (13)	1.4 (0.4−3.8)	2/24 (8)	1.8 (0.2–7.7)
Donkeys				
No contact	211/2,223 (10)	1.0	67/1,399 (5)	1.0
Contact	1/2 (50)	9.5 (0.1–748.8)	0/2 (0)	0 (0–38.7)
Cats				
No contact	203/2,106 (10)	1.0	66/1,315 (5)	1.0
Contact	9/119 (8)	0.8 (0.3–1.5)	1/86 (1)	0.2 (0.05–1.3)
Sheep				
No contact	212/2,218 (10)	1.0	67/1,387 (5)	1.0
Contact	0/7 (0)	0 (0–5.2)	0/14 (0)	0.0 (0–5.5)
Dogs				
No contact	210/2,146 (10)	1.0	67/1,398 (5)	1.0
Contact	2/79 (3)	0.2 (0.03–0.9)	0/3 (0)	0.0 (0–11)
Camels				
No contact	196/2,173 (9)	1.0	67/1,365 (5)	1.0
Contact	16/52 (31)	4.5 (2.3–8.5)†	0/36 (0)	0.0 (0.0–2.1)

*SFG, spotted fever group; STG, scrub typhus group; OR, odds ratio. †Seroprevalence for both spotted fever and scrub typhus increased with age; there were significant differences (p<0.05) between those <5 years of age and those in older age groups for SFG and those >12 years of age for STG. Exposure to SFG and STG were more likely in patients who had contact with dogs and camels for SFG and goats for STG (p<0.05). OR, odds ratio.

## Conclusions

Seventy-eight percent of the study population was >12 years of age; >50% were <5 years of age. This age weighting may have led to underreporting of seroprevalence, because seroprevalence increased with age for SFG and STG (Table). The overall seroprevalence of SFG was 10% (212/2,225), similar to the percentage reported among febrile patients in northern Tanzania (8%) (*10*). Substantial differences in seroprevalence were observed among patients in the surveillance hospitals in different ecoregions of Kenya (Technical Appendix Figure 2). Patients’ land use influenced seroprevalence; the highest rates of seroprevalence were recorded among the pastoralists of Garissa and Marigat, who keep large herds of cattle, sheep, goats, and camels. In other locales with high seroprevalence rates (Alupe, Malindi, Kisii, and Kisumu), farmers practice small-scale animal husbandry. IgG titers in most seropositive patients were high (1,600–6,400), perhaps indicating patients’ repeated exposure to other homologous or heterologous SFG organisms (Figure 1, Panel A). In contrast to seroprevalence of SFG, seroprevalence of TG rickettsioses was low (4/1,611, <1%) and comparable to that reported among febrile patients in northern Tanzania (*10*).

Considering that STG had not been reported in Kenya, its seroprevalence was surprisingly high (67/1,401, 5%) and was highest in Marigat (28/238, 12%) (Technical Appendix Figure 3). Marigat also had the highest number of persons with titers of 1:400 and 1:1,600 (Figure 1, Panel B). The reported determinants for STG are presence of rodents and vectors (chigger mites, especially *Leptotrombidium deliense*) that infest areas of heavy scrub vegetation.

Exposure to scrub typhus increased with age; persons >26 years of age were more likely to be seropositive than younger persons. Similar results were reported in a study conducted in Sri Lanka (*11*). Scrub typhus seropositivity was associated with contact with goats, perhaps because the short dense shrubs that are forage for goats are also the habitat for Trombiculid mites. As in South Korea but not Japan (*12*), more girls and women were exposed to tsutsugamushi disease than boys and men, possibly because women’s culturally sanctioned activities expose them to plant tissues inhabited by chiggers.

This study had several limitations. First, the serum samples used were from a 1-time encounter with the patient (acute-phase sample only). Convalescent-phase serum samples would have better defined the cases. Second, to demonstrate the disease unequivocally, the Trombiculid mites and the infectious *Orientia* spp. will need to be identified. Last, it remains to be determined whether the findings of STG in Kenya represents spread of *Orientia* species outside the tsutsugamushi triangle (an area that includes Pakistan, Australia, Japan, South Korea, and Thailand), as reported recently (*3*,*13*), or identifies a hitherto unknown disease-endemic focus.

**Technical Appendix.** Additional information regarding seroprevalence of IgG against spotted fever group rickettsiae and scrub typhus in patients recruited from different surveillance hospitals in Kenya

## References

[1] Raoult D, Fournier PE, Fenollar F, Jensenius M, Prioe T, de Pina JJ, *Rickettsia africae*, a tick-borne pathogen in travelers to sub-Saharan Africa. N Engl J Med. 2001;344:1504–10. 10.1056/NEJM200105173442003 11357153

[2] La Scola B, Raoult D. Laboratory diagnosis of rickettsioses: current approaches to diagnosis of old and new rickettsial diseases. J Clin Microbiol. 1997;35:2715–27. 9350721 10.1128/jcm.35.11.2715-2727.1997 PMC230049

[3] Izzard L, Fuller A, Blacksell SD, Paris DH, Richards AL, Aukkanit N, Isolation of a novel *Orientia* species (*O. chuto* sp. nov.) from a patient infected in Dubai. J Clin Microbiol. 2010;48:4404–9. 10.1128/JCM.01526-10 20926708 PMC3008486

[4] Richards AL, Jiang J, Omulo S, Dare R, Abdirahman K, Ali A, Human infection with *Rickettsia felis*, Kenya. Emerg Infect Dis. 2010;16:1081–6. 10.3201/eid1607.091885 20587178 PMC3321909

[5] Jiang J, Marienau KJ, May LA, Beecham HJ 3rd, Wilkinson R, Ching WM, Laboratory diagnosis of two scrub typhus outbreaks at Camp Fuji, Japan in 2000 and 2001 by enzyme-linked immunosorbent assay, rapid flow assay, and Western blot assay using outer membrane 56-kD recombinant protiens. Am J Trop Med Hyg. 2003;69:60–6. 12932099

[6] Maina AN, Knobel DL, Jiang J, Halliday J, Feikin DR, Cleaveland S, *Rickettsia felis* infection in febrile patients, western Kenya, 2007–2010. Emerg Infect Dis. 2012;18:328–31. 10.3201/eid1802.111372 22304807 PMC3310467

[7] Mutai BK, Wainaina JM, Magiri CG, Nganga JK, Ithondeka PM, Njagi ON, Zoonotic surveillance for rickettsiae in domestic animals in Kenya. Vector Borne Zoonotic Dis. 2013;13:360–6. 10.1089/vbz.2012.0977 23477290

[8] Richards AL, Soeatmadji DW, Widodo MA, Sardjono TW, Yanuwiadi B, Hernowati TE, Seroepidemiologic evidence for murine and scrub typhus in Malang, Indonesia. Am J Trop Med Hyg. 1997;57:91–5. 9242326 10.4269/ajtmh.1997.57.91

[9] Graf PC, Chretien JP, Ung L, Gaydos JC, Richards AL. Prevalence of seropositivity to spotted fever group rickettsiae and *Anaplasma phagocytophilum* in a large, demographically diverse US sample. Clin Infect Dis. 2008;46:70–7. 10.1086/524018 18171216

[10] Prabhu M, Nicholson WL, Roche AJ, Kersh GJ, Fitzpatrick KA, Oliver LD, Q fever, spotted fever group, and typhus group rickettsioses among hospitalized febrile patients in northern Tanzania. Clin Infect Dis. 2011;53:e8–15. 10.1093/cid/cir411 21810740 PMC3148261

[11] Reller ME, Bodinayake C, Nagahawatte A, Devasiri V, Kodikara-Arachichi W, Strouse JJ, Unsuspected rickettsioses among patients with acute febrile illness, Sri Lanka, 2007. Emerg Infect Dis. 2012;18:825–9. 10.3201/eid1805.111563 22516455 PMC3358078

[12] Bang HA, Lee MJ, Lee WC. Comparative research on epidemiological aspects of tsutsugamushi disease (scrub typhus) between Korea and Japan. Jpn J Infect Dis. 2008;61:148–50. 18362409

[13] Balcells ME, Rabagliati R, Garcia P, Poggi H, Oddo D, Concha M, Endemic scrub typhus–like illness, Chile. Emerg Infect Dis. 2011;17:1659–63. 10.3201/eid1709.100960 21888791 PMC3322051

