# Supplementary material for: High Seroprevalence of Antibodies against Spotted Fever and Scrub Typhus Bacteria in Patients with Febrile Illness, Kenya
Source: Emerg Infect Dis. 2015 Apr;21(4):688–91. doi: 10.3201/eid2104.141387 (PMC4378494; doi:10.3201/eid2104.141387)
Supplement: Supplementary file 1 — Technical Appendix. Additional information regarding seroprevalence of IgG against spotted fever group rickettsiae and scrub typhus in patients recruited from different surveillance hospitals in Kenya [file 14-1387-Techapp-s1.pdf]

Article DOI: http://dx.doi.org/10.3201/eid2104.141387

# High Seroprevalence of Antibodies against Spotted Fever and Scrub Typhus Bacteria in Patients with Febrile Illness, Kenya

## Technical Appendix

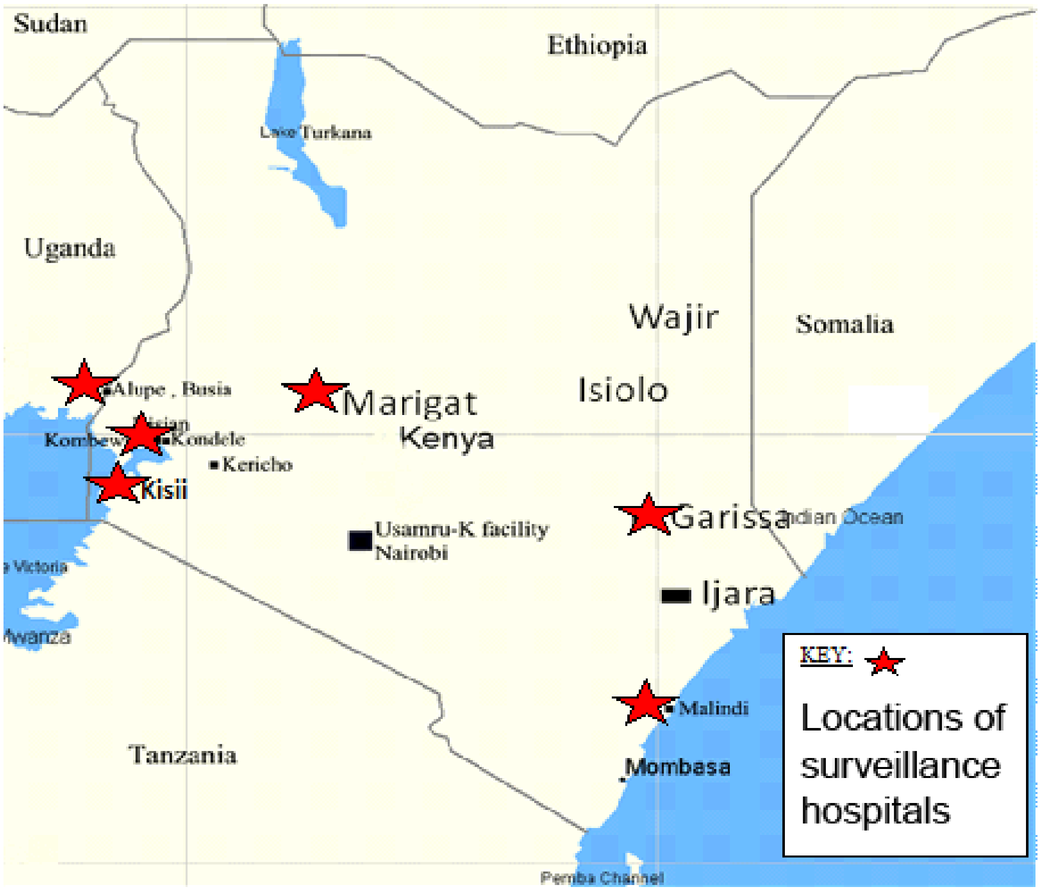


**Technical Appendix Figure 1.** Map of Kenya showing locations of various surveillance hospitals. Lake Victoria basin: Kisumu District Hospital, New Nyanza Provincial Hospital, and Alupe District Hospital. Kisii highlands: Kisii District Hospital. Semiarid, on the floor of the Rift Valley: Marigat District Hospital. Arid northeastern Kenya: Garissa District Hospital and Iftin Sub-District Hospital. Coast of Indian Ocean: Malindi District Hospital.

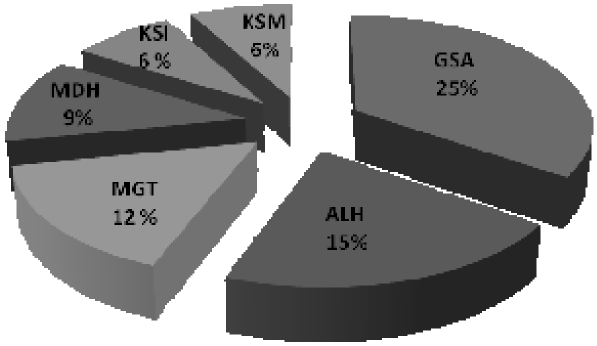


**Technical Appendix Figure 2**. Seroprevalence of IgG against spotted fever group rickettsiae in patients recruited from different surveillance hospitals in Kenya. Garissa District Hospital and Iftin Sub-District Hospital (GSA) in semiarid northeastern Kenya had the highest prevalence compared with Alupe District Hospital (ALH) on the Kenya-Uganda border, Marigat District Hospital (MGT) on the floor of the Rift Valley, Malindi District Hospital (MDH) on the Indian Ocean coast, Kisii District Hospital (KSI) in the Kisii highlands, and Kisumu District Hospital and Obama Children's Hospital (KSM) in the Lake Victoria basin.

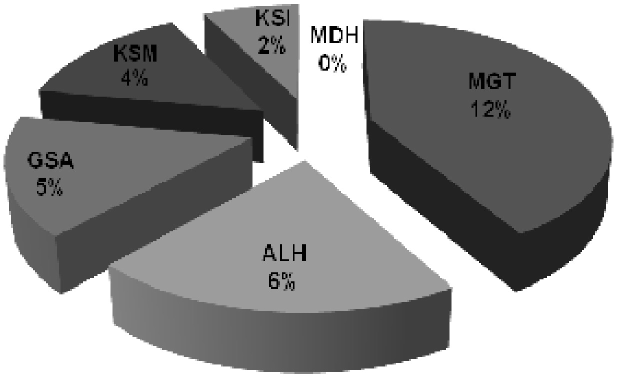


**Technical Appendix Figure 3.** Seroprevalence of scrub typhus in patients recruited from surveillance hospitals. Marigat District Hospital (MGT), on the floor of the Rift Valley, had the highest prevalence compared with Alupe District Hospital (ALH) on the Kenya-Uganda border; Garissa District Hospital and Iftin Sub-District Hospital (GSA) in northeastern Kenya; Kisumu District Hospital and Obama Children's Hospital (KSM) on the Lake Victoria basin; Kisii District Hospital (KSI) in the Kisii highlands; and Malindi District Hospital (MDH) on the Indian Ocean coast.
